# BST2/Tetherin Overexpression Modulates Morbillivirus Glycoprotein Production to Inhibit Cell–Cell Fusion

**DOI:** 10.3390/v11080692

**Published:** 2019-07-30

**Authors:** James T. Kelly, Stacey Human, Joseph Alderman, Fatoumatta Jobe, Leanne Logan, Thomas Rix, Daniel Gonçalves-Carneiro, Corwin Leung, Nazia Thakur, Jamie Birch, Dalan Bailey

**Affiliations:** 1 Viral Glycoproteins Group, The Pirbright Institute, Ash Rd, Guildford, Surrey GU24 0NF, UK; 2 Institute of Immunology and Immunotherapy, The University of Birmingham, Birmingham B15 2TT, UK

**Keywords:** *Morbillivirus*, haemagluttinin, measles, PPRV, MeV, BST2, tetherin

## Abstract

The measles virus (MeV), a member of the genus *Morbillivirus*, is an established pathogen of humans. A key feature of morbilliviruses is their ability to spread by virus–cell and cell–cell fusion. The latter process, which leads to syncytia formation in vitro and in vivo, is driven by the viral fusion (F) and haemagglutinin (H) glycoproteins. In this study, we demonstrate that MeV glycoproteins are sensitive to inhibition by bone marrow stromal antigen 2 (BST2/Tetherin/CD317) proteins. BST2 overexpression causes a large reduction in MeV syncytia expansion. Using quantitative cell–cell fusion assays, immunolabeling, and biochemistry we further demonstrate that ectopically expressed BST2 directly inhibits MeV cell–cell fusion. This restriction is mediated by the targeting of the MeV H glycoprotein, but not other MeV proteins. Using truncation mutants, we further establish that the C-terminal glycosyl-phosphatidylinositol (GPI) anchor of BST2 is required for the restriction of MeV replication in vitro and cell–cell fusion. By extending our study to the ruminant morbillivirus peste des petits ruminants virus (PPRV) and its natural host, sheep, we also confirm this is a broad and cross-species specific phenotype.

## 1. Introduction

As recently as 1980, the measles virus (MeV) killed 2,600,000 people per year, however, the effective use of a live attenuated vaccine has led to a significant drop in fatalities. Unfortunately, MeV remains endemic in many developing countries, causing over 100,000 deaths per year (World Health Organization statistics, [1]). The MeV is a small RNA virus, classified in the genus *Morbillivirus*, which encodes six transcription units and at least eight proteins. Two of these proteins, the fusion (F) and haemagglutinin (H) proteins, are glycoproteins, embedded as functional oligomers in the surface of the viral envelope [2]. H directs attachment to one of two known receptors, SLAMF1 (signalling lymphocyte activation molecule F1) or nectin-4, while F initiates the membrane fusion events required for genome invasion [2,3]. SLAMF1 and nectin-4 are found on separate cells in vivo, immune and epithelial, respectively, contributing to a MeV life cycle that involves infection of both the lymphatic system and various epithelia [3]. One of the characteristic features of a MeV infection is the formation of syncytia, or multinucleated cells, both in vitro and in vivo, i.e., in the lymph node, thymus, and respiratory tract [4,5]. Syncytia formation, a process which occurs when uninfected cells become fused by neighboring, F- and H-expressing infected cells is possible because MeV glycoproteins are functional at neutral pH [6]. Morbilliviruses can also spread by canonical particle formation (viral budding) and both processes contribute to viral dissemination and pathogenesis in the host, however, the mechanisms underpinning the equilibrium between budding and cell–cell fusion are poorly understood.

Although MeV can potently inhibit innate immune signaling, particularly through the action of its accessory protein V [7], there is evidence that a robust type I interferon (IFN) response is mounted in infected cells, both in vivo and in vitro, i.e., in patient peripheral blood mononuclear cells (PBMCs) [8], mouse models [9], and primary dendritic cell cultures [10]. Amongst the upregulated genes observed in IFN-simulated cells are a range of well-characterized interferon stimulated genes (ISGs) that facilitate an antiviral state, including the restriction factor bone marrow stromal antigen 2 *(BST2*) and its resulting protein, known as BST2, Tetherin or CD317.

BST2 is a well-characterized IFN-inducible protein that is capable of restricting the release of a broad range of enveloped viruses [11]. Its principal mechanism of action is to tether nascent virus to the cell surface, preventing effective release [11,12]. The antiviral properties of BST2 were first characterized by Neil et al., who, at the same time, identified a virally encoded antagonist, HIV-1 Vpu [12]. The BST2 protein is capable of restricting both cell-free and cell-cell routes of HIV-1 transmission [13]. Its antagonist, Vpu, in turn, is capable of redirecting this protein away from sites of HIV-1 budding through the hijack of membrane trafficking [14]. Since its discovery, BST2-mediated restriction and viral antagonists have been identified in a range of viruses including Ebola virus [15], hepatitis C virus [16], dengue virus [17], and herpes simplex virus [18]. More recently, BST2 has been characterized as both an important immune sensor, through induction of proinflammatory gene expression via activation of NF-κB [19], and as a modulator, through immunoglobulin-like transcript 7 (ILT7) interactions [20]. These signaling pathways are activated in part by phosphorylation of sequences in the cytoplasmic tail of BST2 that resemble hemi-immunoreceptor tyrosine-based activation motifs (hemITAMs) [19].

Since morbilliviruses, including MeV, frequently remain cell-associated and spread extensively via cell–cell fusion, rather than through cell-free virus, we investigated whether BST2 overexpression was capable of restricting this aspect of the viral life cycle specifically. Using virulent viruses and quantitative cell–cell fusion assays (based on viral glycoproteins from field isolates) we have demonstrated that BST2 from separate mammalian species can restrict morbillivirus cell–cell fusion when overexpressed in cells. This process is dependent on an overall reduction in viral glycoprotein levels, a decrease that is, in part, dependent on the BST2 GPI anchor.

## 2. Materials and Methods

### 2.1. Cells

The HEK293T, HEK293T stably expressing human SLAMF1 (293-hSLAM), and Vero stably expressing human or canine SLAMF1 (Vero-h/cSLAM cells) were maintained in DMEM media containing 10% fetal bovine serum (FBS) at 37 °C with 5% CO_2_. 293-hSLAMcells were generated, as described previously, using a lentivirus transduction system and maintained with 1 μg/mL Puromycin [21]. The Vero-h/cSLAM cells were maintained with 0.4 mg/mL geneticin.

### 2.2. Viruses and Viral RNA Quantification

The MeV-GFP, an EGFP-expressing recombinant MeV (strain IC323), was generated as reported previously [22]. Virus stocks were grown and titred in Vero hSLAM cells. PPRV, Turkey 2000, a wild-type strain was grown in Vero cSLAMcells. Virus titres were calculated by TCID50 using the Reed-Müench method following a single freeze–thaw cycle at −80 °C. Viral genome was detected using a SYBR-based strand-specific RT-qPCR protocol targeting the N gene, using a standard curve for quantification.

### 2.3. Plasmids

The BST2 ORFs from human and ovine genes were amplified by RT-PCR from HEK293T and sheep epithelial cell lines, respectively, using Superscript II Reverse Transcriptase (ThermoFisher, Waltham, MA, USA) and Kod HiFi DNA polymerase (MerckMillipore, Burlington, MA, USA). Primer sequences were designed so as to incorporate an N-terminal FLAG tag peptide sequence into the resultant BST2 protein. MeV and PPRV N, F and H ORFs were amplified by RT-PCR from cells infected with the virulent MeV-IC323, MeV-Dublin, and PPRV-Turkey 2000 virus strains, respectively. All cell–cell fusion assays were performed with MeV-Dublin strain constructs. No significant difference was observed in the BST2-mediated restriction of IC323 or Dublin based cell–cell fusion. The Dublin MeV H expression construct was amplified to include an N-terminal HA-tag sequence. The cDNAs were directionally cloned into the multiple cloning sites of the eukaryotic expression vector pcDNA3.1 (ThermoFisher) under the control of a CMV promoter. The MeV F and H constructs expressing these viral glycoproteins with truncated cytoplasmic tails, were generated as described previously [23,24], by mutagenic PCR and cloned into pcDNA3.1, as were the BST2 mutants ∆-GPI (lacking 19 C-terminal amino acids) and ∆-TM (lacking 46 N-terminal amino acids). Human SLAM was generated by RT-PCR from Vero-hSLAM cells and cloned into a lentivirus expression system, as described previously [21]. All primer sequences and restriction endonuclease cloning strategies are available upon request.

### 2.4. Infections

The 293-hSLAM cells were plated at a density of 1 × 10^5^ cells per well in 24 well dishes. The following day, cells were transfected with 500 ng of plasmid DNA (pcDNA3.1, pcDNA3.1-BST2, pcDNA3.1-∆-GPI or pcDNA3.1-∆-TM) using Transit X-2 transfection reagent (Mirus, Madison, WI, USA) and Optimem (ThermoFisher), as per the manufacturer’s instructions. Twenty-four hours later the media was removed and cells were infected with MeV-GFP at various MOIs (as determined by TCID50) in a 500 μL inoculum volume. After 1 h of incubation at 37 °C the inoculum was removed and fresh media was added to the cells. At various times post infection, the supernatant from infected cells was removed and frozen (to quantify released virus). Fresh media was then added to the remaining cells and these were frozen to quantify the cell-associated virus. All experiments were carried out with biological triplicates. For phase-contrast microscopy, MeV-GFP infected cells were visualized by phase-contrast microscopy using an inverted UV microscope (Nikon Eclipse TE2000-5 microscope coupled with a Nikon HB-10101AF super high-pressure mercury lamp) equipped with a Hamamatsu C472-95 digital camera (Sony, Minato-ku, Japan).

### 2.5. Pseudotyped Viruses

HEK293T cells were plated at a density of 7.5 × 10^5^ cells per well in 6 well dishes. The following day they were transfected with 3.5 µg each of pcDNA3.1 constructs expressing MeV F and H with 30 and 24 amino acid cytoplasmic tail truncations, as well as 1.5 µg of p8.91 (encoding for HIV-1 gag-pol) and 1 µg of CSFLW (the luciferase reporter-expressing lentivirus-backbone). Supernatants containing pseudotyped virus (MeV-PP) were harvested at 72 h post transfection, clarified by centrifugation, and frozen to −80 °C. The target 293-hSLAM cells were plated at a density of 1 × 10^5^ cells per well in 24 well dishes one day prior to transduction/infection for 72 h. Firefly luciferase activity in these cells was assayed using the Luciferase Assay System (Promega, Madison, WI, USA) according to the manufacturer’s instructions and a Promega GloMax multimode plate reader.

### 2.6. Fusion Assays

The HEK293T cells were plated out to a cell density of 7.5 × 10^5^ cells per well in 6 well dishes. The following day, effector cells were transfected with 500 ng each of MeV or PPRV F and H expression constructs, 500 ng of the 1–7 fragment of rLuc-GFP [25] and 1 μg of either the blank vector control (pcDNA3.1) or a BST2 expression vector (as indicated). Separately, target cells were transfected with 1 μg of lentivirus vectors expressing human or ovine SLAMF1, as well as 500 ng of the 8–11 fragment of rLuc-GFP. All transfections were performed using TransitX transfection reagent (Mirus), according to the manufacturer’s instructions. Following 48 h of incubation, effector and target cells were washed, counted, and co-cultured at a ratio of 1:1 in white-walled 96 well plates to a final density of 1 × 10^5^ cells per well. Then, 16–24 h later the Renilla luciferase activity in fused cells was measured (in a Promega GloMax multi-mode plate reader) by removing the media and adding 2 μg/mL of cell-permeable coelenterazine 400A (Biotium, Fremont, CA, USA), in PBS. Normally, five or more co-culturing replicates were performed for each biological condition.

### 2.7. Incucyte Fluorometric Quantification

GFP fluorescence, including total GFP intensity and average green object size, were quantified using an Incucyte S3 real-time imager (Essen Bioscience, Ann Arbor, MI, USA) with cells being maintained under the same conditions listed previously (37 °C with 5% CO_2_). Phase images were captured regularly and masking applied to identify individual cells. Concurrently, GFP fluorescence was quantified using a built-in fluorescence detection filter.

### 2.8. Protein Labeling and Quantification

To examine protein co-expression, 293-hSLAM cells were plated at a density of 1 × 10^5^ cells per well in 24 well dishes, transfected with relevant combinations of BST2 and viral protein expression constructs using Transit X2 transfection reagent (Mirus), and lysed at 16–24 h post transfection. All protein samples were generated in 1X radio-immunoprecipitation assay (RIPA) buffer containing protease inhibitors (ThermoFisher). Briefly, existing growth media was removed and cells were washed in phosphate buffered saline (PBS) before being pelleted by centrifugation. Pelleted cells were then resuspended in 1X RIPA and left on ice for 10 min before repeated centrifugation at high speed (16,000× *g*) for a further 10 min at 4 °C. Protein lysate-containing supernatants were then stored at −20 °C until required. Samples for western blot were analyzed by SDS-PAGE, semi-dry, PVDF-based, transfer, and blotting in TBS-Tween containing 5% (*w/v*) milk powder. All primary antibodies were incubated overnight at 4 °C. The following antibodies were used: anti-MeV nucleocapsid (N505) and anti-morbillivirus/MeV haemagglutinin (cytoplasmic tail) (rabbit polyclonal at 1:1000, gifted from R. Cattaneo, [26]), anti-FLAG (1:1000, Cell Signaling (CS), 9A3), anti-GAPDH (1:1000, 14C10, CS), anti-HA (1:1000, CS, C29F4), anti-tubulin (1:1000, CS, 9F3) and standard HRP-linked secondary antibodies (CS). For flow cytometry analysis of transfected 293-hSLAMs, cells were immunolabeled in PBS with 1% BSA, 0.01% NaN_3,_ and protease inhibitors (ThermoFisher) together with the PE-conjugated anti-SLAMF1 antibody (BD, 559592, 1:100). Labeled, or isotype-control labeled cells, were then fixed in a solution containing 2% PFA, PBS, and 0.01 % NaN_3_ and cells were analyzed using a CyAn Analyzer flow cytometer (Beckman Coulter, Brea, CA, USA). Following appropriate gating the mean fluorescence intensity of SLAMF1 positive cells was calculated from triplicate analyses. For immunofluorescence analysis by confocal imaging transfected Vero hSLAM cells (24 h post transfection) were fixed in 4% PFA PBS, permeabilized in 0.2% TX-100 PBS, and blocked and stained in 1% BSA PBS. The antibodies used for staining were anti-FLAG (CS, 1:100), anti-HA (CS, 1:100), anti-PPRV H (C77 mAb, 1:100) and standard fluorophore-conjugated secondary antibodies (Invitrogen, Carlsbad, CA, USA). Slides were mounted using Mowiol mounting medium (Merck Millipore) containing Hoescht 33342 DNA stain. To visualize the cells we used a Leiss LSM 510 Meta Confocal Microscope.

### 2.9. Phylogenetic Analysis

A comparison of BST2 amino acid sequences was performed using the Vector Nti package (ThermoFisher), particularly the AlignX embedded software. The sequences analyzed were as follows: XP_006747308 *Leptonychotes weddellii* (seal), XP_865603 *Canis lupus familiaris* (dog), NP_001230014 *Felis catus* (cat), NP_004326 *Homo sapiens* (human), XP_004277750 *Orcinus orca* (whale), NP_001171522 *Ovis aries* (sheep, BST2B), DAA28235 *Bos taurus* (cow), and NP_001171521 *Ovis aries* (sheep, BST2A).

### 2.10. Statistical Analysis and Data Handling

All experimental data sets contain a minimum of three biological replicates. Statistical analysis was performed using an unpaired, one-tailed t-test (*, *p* < 0.05; **, *p* < 0.005; ***, *p* < 0.0005; ****, *p* < 0.0001) within the GraphPad Prism file.

## 3. Results

### 3.1. MeV Replication is Sensitive to BST2 Overexpression

**Figure 1:**
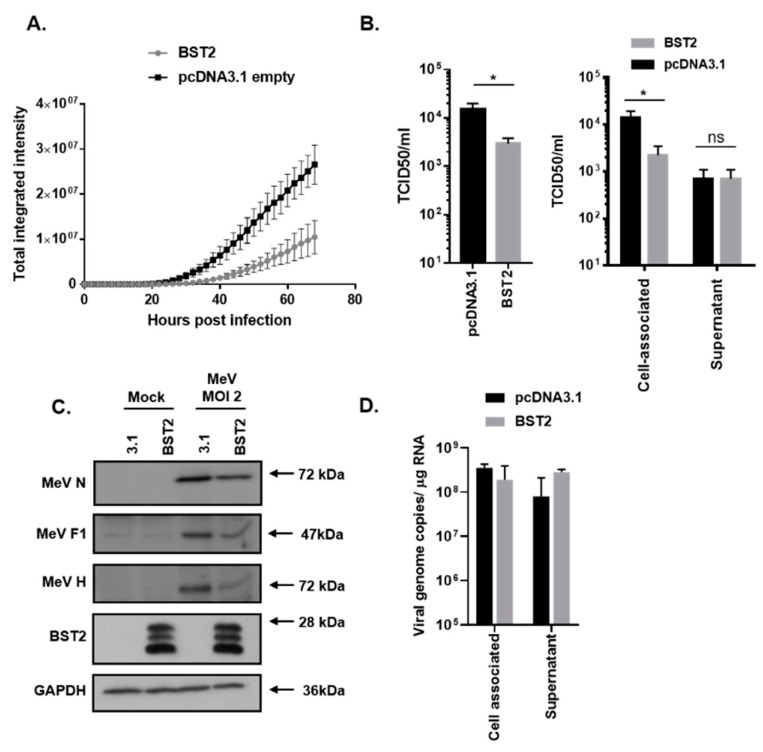
Measles virus (MeV) replication is sensitive to bone marrow stromal antigen 2 (BST2). 293-hSLAMs, transfected with the indicated pcDNA3.1-based expression construct for 24 h, were infected with MeV-GFP at a multiplicity of infection (MOI) of 2 (**A–D**) and virus replication assayed periodically using an Incucyte imager for 72 h with GFP as a marker (**A**). In equivalent experiments mean, virus titres were calculated at 48 h post infection by TCID50 analysis of triplicate biological samples of supernatant and cell-associated virus (**B**); (left panel, sum of total virus titres and right panel, individual fractions). At equivalent time points infected cells were lysed in RIPA buffer and analyzed using western blot to quantify viral protein production (**C**) or, separately, the RNA was extracted, and the genome was quantified using strand-specific qPCR (**D**). Error bars indicate standard deviation of the mean. Statistical significance is as follows: *, *p* < 0.05.

To analyze MeV restriction by BST2, a permissive (to both infection and transfection) cell line was generated as follows: The HEK293T cells were engineered to overexpress human SLAMF1 (293-hSLAM) using a standard lentivirus-based transduction system as described previously [21], with receptor expression being confirmed by flow cytometry. 293-hSLAMs, transfected with a FLAG-tagged pcDNA3.1-BST2 expression construct (Figure 1, pcDNA3.1-BST2/BST2) or pcDNA3.1 mock control plasmid (Figure 1, 3.1) for 24 h, were subsequently infected at high (2) multiplicity of infection (MOI) with a recombinant MeV, expressing green fluorescent protein (GFP) as a separate transcription unit (MeV-GFP) [22]. Using GFP as a marker of viral replication and an Incucyte real-time plate imager to quantify fluorescence we identified a significant inhibition in MeV-GFP replication when BST2 was expressed in cells (Figure 1A). Infectious virus was then quantified at 48 h post infection by tissue culture infectious dose 50 (TCID50) titration, identifying an 80% reduction in virus yields when BST2 was overexpressed (Figure 1B). As expected, the majority of the virus detected was cell-associated as is typical of MeV infections; however, interestingly, no difference was seen in the levels of infectious virus released from cells, although these titres were low (<10^3^/mL) (Figure 1B, supernatant). This reduction in MeV-GFP replication was supported by an observed reduction in MeV nucleocapsid (N), F, and H proteins, as detected by MeV-specific antibodies and western blot, with, respectively, a 41%, 75%, and 80% reduction in each viral protein observed when the bands were analyzed by densitometry (Figure 1C). A typical expression pattern for BST2, indicative of multiple glycosylation of this protein, was also seen, whereas, no change to the endogenous control GAPDH (Figure 1C) was seen. To further examine the effect of BST2 overexpression on MeV-GFP release we quantified viral RNA in the cell-associated and supernatant fractions, identifying a clear difference between infectious virus (Figure 1B) and viral RNA present in these fractions (Figure 1D), when comparing BST2-expressing cells to mock pcDNA3.1 transfected cells. Specifically, while there was no clear difference between infectious virus yields in the supernatant of pcDNA3.1 and BST2-transfected cells, there was more viral RNA in the latter, indicating potential modification of particle infectivity. These data indicate that MeV replication is sensitive to BST2 expression, however, this does not appear to be due to inhibition of MeV egress into the supernatant.

### 3.2. MeV Entry is Not Inhibited by BST2

One interpretation of our data from infected 293-hSLAMs is that MeV entry, specifically, is inhibited by ectopic expression of BST2 in these cells. To examine MeV entry we used a luciferase reporter-expressing, replication-incompetent, HIV-1 pseudotyped with MeV glycoproteins F and H (MeV-PP) [23,24]. The 293-hSLAM cells were transfected with the BST2 expression construct or the pcDNA3.1 control vector for 24 h, and, subsequently, infected with MeV-PP for 48 h. No significant difference in MeV-PP entry was observed when comparing the two conditions (Figure S1A). We also examined whether expression of SLAMF1, in our stable 293-hSLAM cell line, was affected by BST2 expression. However, using flow cytometry to assess surface SLAMF1, we again saw no effect (Figure S1B). Collectively, these data indicate that MeV entry and SLAMF1 expression are not inhibited by expression of BST2 in permissive cells.

### 3.3. MeV Cell–Cell Fusion is Inhibited by BST2

**Figure 2:**
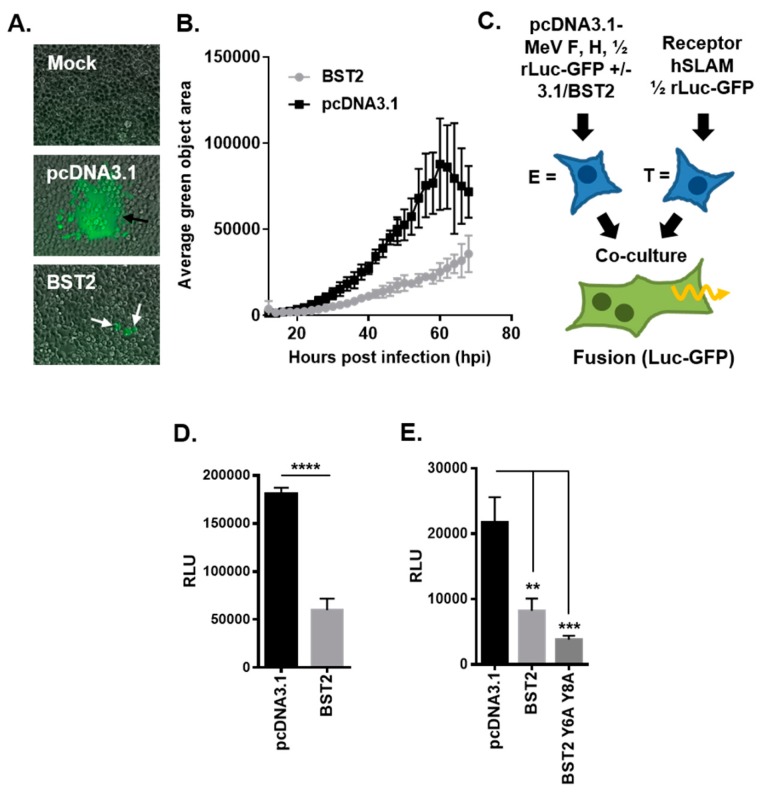
MeV cell–cell fusion is inhibited by BST2. (**A/B**) The average green object size (syncytia) in high MOI (2), MeV-GFP infected 293-hSLAM cells, transfected with the indicated pcDNA3.1-based expression constructs, was quantified at the indicated times post infection using an Incucyte imager. (**A**) Representative micrographs taken at 24 h post infection (black arrows, typical syncytia in pcDNA3.1 transfected; white arrows, smaller foci with pcDNA3.1-BST2). (**C**) The MeV cell–cell fusion assay involves separate transfection/treatment of effector E and target T populations prior to co-culturing, as indicated. Fused cells can be visualized and quantified due to reconstitution of the rLuc-GFP reporter. (**D**) The HEK293T MeV effector cells, expressing MeV fusion (F) and haemagglutinin (H), were cotransfected with the indicated pcDNA3.1-based expression constructs before cell fusion was quantified (by assaying *Renilla* luciferase activity 16 h after co-culturing with hSLAMF1-positive target cells). (**E**) The MeV F/H effector cells were cotransfected with pcDNA3.1, BST2 wt, or BST2 Y6,8A expression constructs and cell–cell fusion assayed, as described previously. Error bars indicate standard deviation (B) or error (D/E) of the mean. Statistical significance is as follows: **, *p* < 0.005; ***, *p* < 0.0005; ****, *p* < 0.0001.

One striking observation from our BST2 transfection and MeV-GFP infection experiments was a reduced number of large GFP-positive syncytia in BST2 transfected cells as compared with the pcDNA3.1 control (Figure 2A). Although the number of infectious foci was similar, supporting our conclusions that BST2 does not affect MeV entry, these foci (Figure 2A, white arrows) did not frequently develop into large syncytia (Figure 2A, black arrows). Quantification of average syncytia size (calculated as average green object area using the Incucyte software, for more details see Figure S2), confirmed this observation (Figure 2B) indicating a specific effect of BST2 on cell–cell fusion. Using an adapted cell–cell fusion assay [25], based on the combination of a dual split *Renilla* luciferase (rLuc) and GFP reporter and independent expression of MeV GPs and human SLAMF1 on effector and target cells, respectively (see schematic, Figure 2C) we were able to assess whether BST2 can inhibit MeV cell–cell fusion. In these assays, effector cells are cotransfected with Mev F and H and one half of the rLuc-GFP reporter. After 48 h of incubation these effector cells are co-cultured with HEK293T cells transfected with a human SLAMF1 expression construct and the other half of the rLuc-GFP reporter. To assess BST-specific effects, pcDNA3.1-BST2 or pcDNA3.1 were additionally cotransfected with MeV F and H into effector cells. Co-expression of BST2 significantly inhibited MeV-induced cell–cell fusion in this assay, as determined by the activity of rLuc when using a cell permeable live-cell substrate (Figure 2D). Importantly, we confirmed that the rLuc-GFP reporter protein expression and activity was not affected by BST2 overexpression (Figure S3). In addition, BST2 restriction of cell–cell fusion did not correlate with prior activation of NF-κB in effector cells, since a NF-κB signaling-deficient BST2 mutant (Y6,8A) [19,27] inhibited cell–cell fusion to a similar degree as the wild type (wt) protein (Figure 2E). Modeling the entry and exit stage of the viral life cycle and analyzing its inhibition by BST2 supported a hypothesis that MeV vGPs were being specifically targeted by BST2.

### 3.4. The MeV Haemagglutinin Glycoprotein is Targeted by BST2

**Figure 3:**
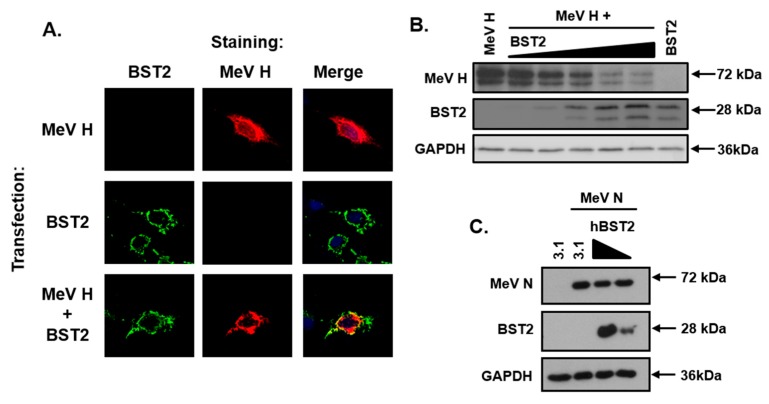
The MeV haemagglutinin glycoprotein is targeted by BST2. (**A**) Vero-hSLAM cells, cotransfected with the indicated pcDNA3.1-based expression constructs, and analyzed by immunofluorescence microscopy. Representative micrographs are shown together with merged images to demonstrate colocalization of labeled proteins. (**B/C**) 293-hSLAMs were transfected with 500 ng of pcDNA3.1-MeV nucleocapsid (N) or H and either pcDNA3.1, or, increasing amounts of pcDNA3.1-BST2 (ranging from 0 to 1.6 μg) and analyzed by western blot. The total mass of transfected DNA remained constant.

To assess whether BST2 targets MeV GPs we focused on the attachment protein, H. A HA-tagged pcDNA3.1-MeV H was transfected into Vero-hSLAM cells with, or without, pcDNA3.1-BST2 and the subcellular localization analyzed by immunofluorescence microscopy. A specific colocalization between BST2 and MeV H was observed in cotransfected cells (Figure 3A). Strikingly, however, the number of MeV H positive cells, visible by fluorescence microscopy during this experiment, was greatly reduced after co-expression with BST2 with only isolated cells expressing detectable levels of both BST2 and H. Subsequent western blot analysis of cotransfected (MeV H and BST2) 293-hSLAM cells demonstrated a BST2 dose-dependent reduction in MeV H expression 16 h after transfection (Figure 3B). This observation appeared specific to MeV H, since experiments examining co-expression of an unrelated viral protein MeV N, the nucleocapsid protein, and BST2 in 293-hSLAM cells showed no equivalent reduction in N protein levels (Figure 3C).

### 3.5. The BST2 GPI Anchor is Required for Restriction of MeV Replication

**Figure 4:**
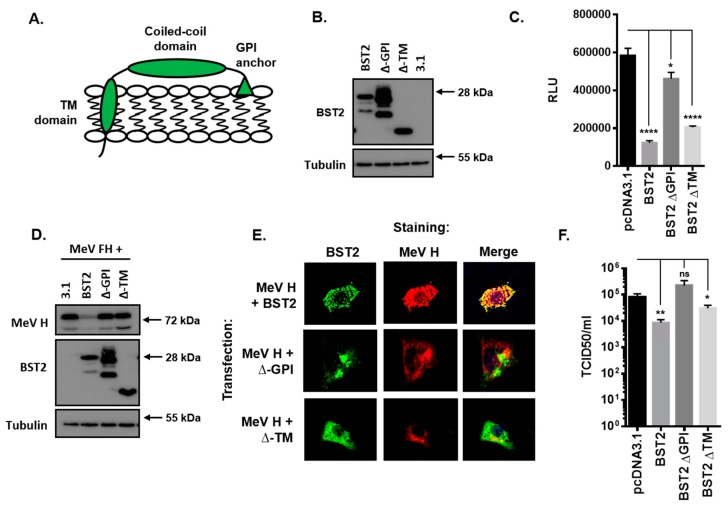
The BST2 GPI anchor is required for restriction of MeV replication. (**A**) The human BST2 protein is a membrane anchored protein composed of various domains (TM, transmembrane; GPI, glycosyl-phosphatidylinositol). (**B/D**) 293-hSLAMs transfected with pcDNA3.1, BST2, ∆-GPI, or ∆-TM were cotransfected with either 500 ng of pcDNA3.1 (**B**) or 250 ng each of pcDNA3.1-MeV F and H (**D**) and analyzed by western blot. The total mass of transfected DNA remained constant. (**C**) The HEK293T MeV effector cells were cotransfected with the indicated pcDNA3.1-based expression constructs and cell fusion quantified by assaying Renilla luciferase activity 16 h after co-culturing with hSLAMF1-positive target cells. (**E**) The Vero-hSLAM cells, cotransfected with the indicated pcDNA3.1-based expression constructs, and analyzed by immunofluorescence microscopy. Representative micrographs are shown together with merged images to demonstrate colocalization of labeled proteins. (**F**) 293-hSLAMs, transfected with the indicated pcDNA3.1-based expression construct for 24 h, were infected with MeV-GFP and total virus replication assayed at 72 h. Error bars indicate standard deviation (F) or error (C) of the mean. Statistical significance is as follows: *, *p* < 0.05; **, *p* < 0.005; ****, *p* < 0.0001.

BST2 is a well-characterized protein with an N-terminal cytoplasmic tail and transmembrane domain, a central coiled-coil domain and a C-terminal glycosyl-phosphatidylinositol (GPI) anchor (Figure 4A). To establish which of these domains are required for restriction of MeV cell–cell fusion and the associated reduction in MeV H protein we generated the following two truncation mutants: A C-terminal mutant lacking the GPI-anchor sequence (∆-GPI) and an N-terminal mutant lacking the cytoplasmic tail and transmembrane domains (∆-TM). The efficient expression of these FLAG-tagged proteins in HEK293T cells was confirmed by western blot (Figure 4B) before application in MeV cell–cell fusion assays. Co-expression of ∆-TM or full length protein (BST2) with MeV F and H in HEK293T effector cells led to a significant reduction in MeV-induced cell–cell fusion after co-culturing with SLAMF1-positive target cells (Figure 4C); however, this inhibition was almost completely absent when the ∆-GPI mutant was used (Figure 4C). Western blot analysis of similarly transfected 293-hSLAM cells demonstrated, as described previously, that full-length BST2 reduced overall MeV H expression as compared with the pcDNA3.1 transfected control; however, interestingly, neither the ∆-GPI nor ∆-TM had any effect on H (Figure 4D). To investigate these two mutants in more detail we, subsequently, analyzed their subcellular localization by immunofluorescence staining of cotransfected Vero-hSLAM cells. The clear colocalization of MeV H with BST2 was lost with both the ∆-GPI and the ∆-TM mutants (Figure 4E), indicating a significant modification in protein function and trafficking for these two truncations, in particular, the ∆-TM mutant of BST2 which appeared to redistribute to the cell surface (Figure 4E). Finally, we assessed whether these mutants also affect infectious MeV replication by performing a BST2-transfection, MeV-GFP infection experiment in permissive 293-hSLAM cells. The restriction of MeV replication by BST2, or the two mutants, was assayed by TCID50 at 72 h post infection and demonstrated that removal of the GPI anchor from BST2 was sufficient to alleviate restriction of infectious MeV (Figure 4F). Altogether, these data demonstrate that the GPI anchor of BST2 is essential for its antagonism of MeV cell–cell fusion and the MeV H protein.

### 3.6. Morbilliviruses are Broadly Targeted by Mammalian BST2 Proteins

**Figure 5:**
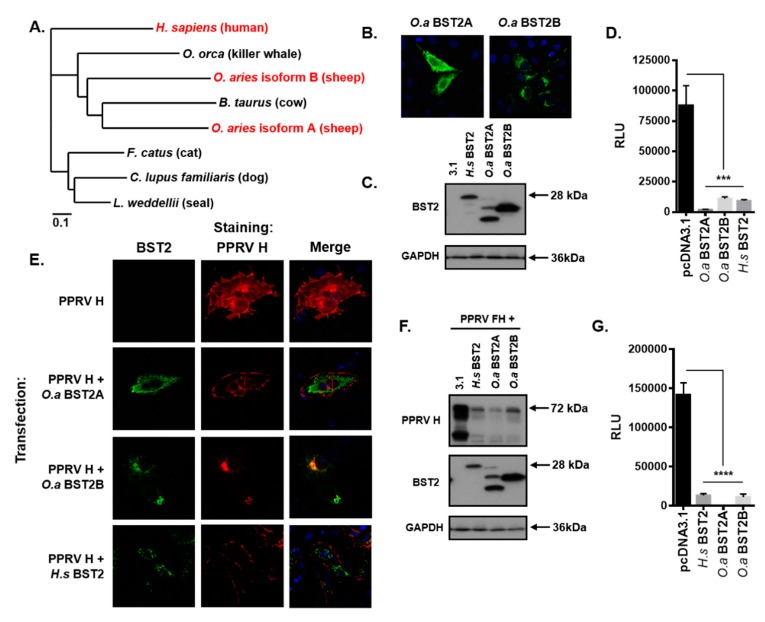
Morbilliviruses are broadly targeted by mammalian BST2 proteins. (**A**) Phylogenetic analysis of the amino acid sequences of a range of mammalian BST2 proteins including those examined in this study (highlighted in red). (**B/E**) Vero-hSLAM cells, cotransfected with the indicated pcDNA3.1-based expression constructs, and analyzed by immunofluorescence microscopy. Representative micrographs are shown together with merged images to demonstrate colocalization of labeled proteins. (**C/F**) HEK293T cells transfected with pcDNA3.1-, *H.s* BST2, *O.a* BST2A, or *O.a* BST2B, were cotransfected with either 500 ng of pcDNA3.1 (**C**) or 250 ng each of pcDNA3.1 peste des petits ruminants virus (PPRV) F and H (**F**) and analyzed by western blot. The total mass of transfected DNA remained constant. (**D/G**) HEK293T PPRV (**D**) or MeV (**G**) effector cells were cotransfected with the indicated pcDNA3.1-based expression constructs and cell fusion quantified by assaying Renilla luciferase activity 16 h after co-culturing with ovine or human SLAMF1-positive target cells, respectively. Error bars indicate standard error of the mean. Statistical significance is as follows: ***, *p* < 0.0005; ****, *p* < 0.0001.

To address the specificity of BST2 restriction across the *morbillivirus* genus, and their respective hosts, we focused on the prevalent morbillivirus peste des petits ruminant virus (PPRV) and its small ruminant host, *Ovis aries* (sheep). As reported previously [28], the *BST2* gene is duplicated in sheep, although the longer isoform (BST2B) is significantly different to the shorter protein (BST2A) which was a feature that was evident following phylogenetic analysis of a range of BST2 proteins from morbillivirus-susceptible mammals (Figure 5A). Using RT-PCR, we amplified the ORFs of the *BST2A* and *BST2B* genes and generated FLAG-tagged expression constructs in the pcDNA3.1 expression vector. The expression of these proteins was confirmed by immunofluorescence microscopy (Figure 5B) and western blot (Figure 5C), which confirmed previous observations from Murphy et al., i.e., that these two proteins have different subcellular localizations and molecular characteristics [28]. The ability of these proteins to inhibit PPRV cell–cell fusion was then assessed using our standardized HEK293T assay, replacing the MeV glycoproteins and human SLAMF1 with PPRV F and H (pcDNA3.1-based) expression constructs and the ovine SLAMF1 receptor [21]. Both isoforms significantly inhibited PPRV fusion (Figure 5D), as did the human BST2 protein, indicating effective restriction of morbilliviruses across host species. The subcellular localization of PPRV H and both ovine and human BST2 was then assessed by immunofluorescence microscopy of transfected Vero-hSLAM cells. In all instances the expression of PPRV H in cotransfected cells was lower than in singularly transfected cells (Figure 5E); however, in those instances where co-expression could be identified we only saw significant colocalization between PPRV H and the sheep BST2B isoform. The subcellular localization of PPRV H in these cells was markedly different from the singularly transfected controls, appearing perinuclear and punctate (Figure 5E). Subsequent investigation of cotransfected HEK293T cells by western blot showed that the expression of PPRV H was reduced when either sheep or human BST2 was co-expressed (Figure 5F), similar to our previous results with MeV H (Figure 3). Finally, we addressed whether MeV cell–cell fusion was also sensitive to cross-species BST2 restriction by performing a MeV fusion assay in HEK293T cells. Cotransfection with constructs expressing either sheep or human BST2 significantly reduced the fusion capacity of MeV effector cells, when compared to mock transfected (pcDNA3.1) cells (Figure 5G). These data demonstrate that morbillivirus H proteins are sensitive to broad restriction by mammalian BST2 proteins and that this reduces the capacity of vGP-expressing cells to induce fusion of receptor-bearing target cells.

### 3.7. H Distribution is Modified in BST2-Transfected PPRV-Infected Cells

**Figure 6:**
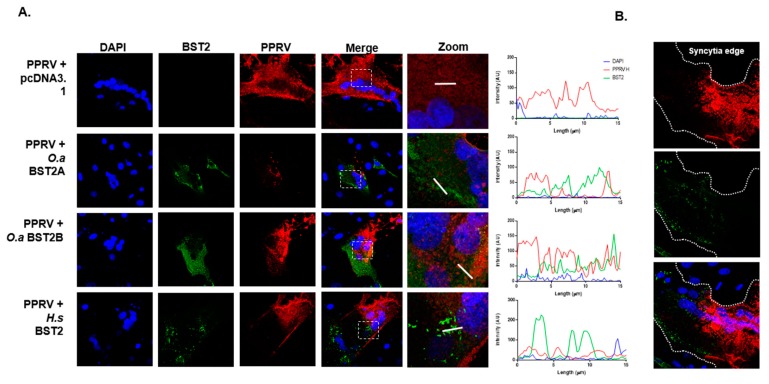
H protein production and trafficking is inhibited in PPRV-infected BST2-overexpressing cells. PPRV H distribution is modified in BST2-transfected infected cells. (**A**) Vero cSLAM cells were transfected with plasmids expressing the indicated BST2 protein (or mock transfected) before being infected with PPRV Tu00 at an MOI of 0.1. Cells were fixed at 16 h post infection before staining with relevant antibodies (PPRV H, red and BST2, green). Micrographs were taken on a Leica confocal microscope with line of interest analysis of colocalization performed using the inbuilt software. The white boxed insets in the merged panels reflect the region chosen for analysis in the “zoom” panel (right), with the x-axis on the colocalization graphs representing the 15 µm white lines indicated on the adjacent zoomed images. (**B**) A zoomed image of a single large BST2-expressing syncytia indicating the approximate position of the plasma membrane.

Finally, we investigated the subcellular distribution of H and BST2 in infected cells. The Vero cells stably expressing canine SLAMF1 (Vero-cSLAM) cells were first transfected with pcDNA3.1 or BST2 expression constructs before being infected with PPRV (Turkey 2000 field strain) at a low MOI (0.1). In the mock-transfected cells, the distribution of PPRV H, within syncytia, was diffuse throughout most of the cytoplasm, with some clustering at the cell surface. However, in syncytia that had been transfected with either human or ovine BST2 there was clear perturbation of H distribution in areas of high BST2 expression (Figure 6A). Zoomed analysis of these images accompanied by quantitative colocalization analysis, together with higher magnification micrographs of syncytia clearly illustrated that within infected cells there was very little colocalization of BST2 and PPRV H and that the trafficking, abundance and/or stability of the viral protein was affected (Figure 6A, zoomed images of boxed insets with accompanying line-of-interest colocalization analysis, and Figure 6B).

## 4. Discussion

Our observation that mammalian BST2 proteins target morbillivirus haemagglutinin proteins contrasts with both the established restriction mechanism of BST2 (in tethering nascent virus to the cell surface [11]) and more recent observations that many vGPs, e.g., Ebola GP [15,29,30], HIV-2 Env [31], and HSV-1 gM [32], have evolved as direct BST2-antagonists. Importantly, the mechanisms underpinning vGP-mediated inhibition of BST2 are likely to be evolutionarily distinct, since both HSV-1 gM and HIV-2 Env have a Vpu-like mechanism for sequestration of BST2 from the cell surface [31,32], while Ebola virus GP is thought to use an alternative approach reliant on the concerted action of its glycan cap and membrane-spanning domain [29,30]. This complexity is also evident when comparing HSV-1 and HSV-2 that are related viruses that have evolved separate mechanisms for vGP restriction of BST2 [32,33]. Interestingly, our results suggest that morbillivirus vGPs have not evolved any BST2-antagonistic phenotype and are actually, in direct contrast, sensitive to inhibition by this protein, particularly, evident in our fusion assays and infected cells. Our data indicate that this inhibition is due to colocalization of BST2 and morbillivirus GPs in intracellular compartments, a reduction in H expression at the cell surface, and, lastly, a BST2 dose-dependent reduction in overall H protein levels. Although Narkpuk et al. observed a similar BST2-mediated down-regulation of transient protein expression in cells [34], the reduction in H protein we observed was specific to this vGP, since neither the viral N protein nor split rLuc-GFP reporter were affected by transient BST2 co-expression. In addition, the trafficking and surface expression of the stably expressed and extensively glycosylated SLAMF1 was also not affected by BST2 expression, which is further indicative of a specific interaction between BST2 and morbillivirus H. Finally, the inhibition of fusion did not appear to correlate to BST2 overexpression induced activation of NF-κB, since the phosphorylation-signaling deficient tyrosine mutant Y6,8A was still capable of inhibiting fusion.

Targeted mutational analysis of BST2 demonstrated that inhibition of MeV fusion and replication is reliant on this protein’s GPI anchor which is consistent with this domain being important in viral restriction [11]. It is important to highlight, however, that although the ∆-TM BST2 mutant was efficient at inhibiting MeV cell–cell fusion it did not reduce the overall level of H protein, unlike the full-length BST2 protein. This intriguing observation, in combination with the altered cellular localization of MeV H when co-expressed with this mutant points to a bipartite effect of BST2 on H, both at the level of cellular trafficking and, potentially, protein degradation. Although the ultimate fate of morbillivirus H proteins remains unclear at this juncture, we hypothesize that BST2 overexpression targets these proteins for proteosomal degradation and this is an area of continued work in our laboratory.

What specifically makes morbillivirus H proteins a target for BST2 is still not known. Since trimers of F and tetramers of H must fold and preassemble as functional oligomers during intracellular trafficking, it is attractive to postulate that morbillivirus-specific aspects of this process are targeted by BST2. One clue to support this hypothesis is that MeV vGPs do not pseudotype well onto lentiviruses, such as the defective HIV-1 system commonly used in laboratories [23,24]. We hypothesize that this occurs because MeV buds from different plasma membrane micro-domains to HIV-1, a process governed by the cytoplasmic tails of MeV vGPs. Accordingly, the defect in MeV vGP pseudotyping is overcome through removal of their cytoplasmic tails [23,24,35]. BST2 may, therefore, have evolved to inhibit virus budding at only specific domains of the cell surface. This hypothesis is strengthened by our observation that MeV genome levels were higher in the supernatant from BST2-transfected cells, although the infective particle number was the same, indicating a perturbation of the normal processes occurring during MeV budding. However, an alternative explanation is the leakage of viral genomes from infected BST2-expressing cells, as there is no direct evidence the detected genomes are from enveloped particles.

This hypothesis, in turn, relates to the absence of significant restriction of MeV release that we observed in our experiments, which was a surprising result given the broad specificity and mechanism of action of this restriction factor [11]. Although it has previously been demonstrated that BST2 can inhibit MeV replication in vitro, these studies quantified only the released virus in the supernatant and did not correlate this to either cell-associated virus yields or the expression of viral proteins and BST2 [9]. Regardless, the observation that released virus was significantly affected by BST2 expression is interesting and markedly contrasts with our own findings. This discrepancy may relate, in part, to the virus strain and receptor used. While we used the virulent IC323 MeV strain and HEK293T cells overexpressing the natural SLAMF1 receptor, Holmgren et al., in 2015 used the attenuated Edmonston vaccine strain which has an extended receptor tropism (binding CD46, in addition to wild-type receptors SLAMF1 and nectin-4) and demonstrably defective innate immune antagonists, especially the accessory protein V that blocks interferon signaling [9,36,37]. Interestingly, the V protein from human parainfluenza virus type 2 (hPIV-2) has recently been shown to interact directly with BST2 to antagonize hPIV-2 restriction [38]. This interaction, specific to C-terminal Tryptophan (Trp) residues in V and the GPI anchor of BST2, leads to re-localization of BST2 from the cell surface without apparent degradation [38]. Since the MeV V protein has a conserved a Trp-containing C-terminal domain [39], it may also be capable of an equivalent restriction of BST2. These putative interactions, as well as a comparison of IC323 and Edmonston V proteins, may explain the strain-specific effect of BST2 on MeV egress and are the focus of ongoing work in our laboratory. Strain-specific interactions with BST2, particularly between lab-adapted and virulent-strain viral proteins have been reported elsewhere, i.e., the HA of multiple influenza virus A strains were shown to variably antagonize BST2 [40], indicating these observations might not be limited to accessory proteins such as V.

Although the broad specificity of mammalian BST2s was evident from our studies, the mechanism of restriction varied, dependent on both the BST2 sequence and target protein. While we observed colocalization between MeV H and *H. sapiens* BST2 this was less evident for PPRV H and the shorter BST2A isoform of *O. aries*. However, a more specific colocalization between PPRV H and BST2 was seen with the longer B isoform, in keeping with studies from Murphy et al., which demonstrated BST2B-specific sequestration of Jaagsiekte sheep retrovirus (JSRV) Env protein into the Golgi apparatus [28]. The duplication of the *O. aries BST2* gene has been reported previously [28,41], in particular, the absence of N-linked glycosylation sites and GPI-anchor sequences in the longer B isoform, characterizations that are supported by our own western blot analysis of this protein. While this points to varying mechanisms of restriction, it should be highlighted that in all cases, including examples of cross-species restriction (e.g., MeV H and *O. aries* BST2), a gross reduction in H protein was seen. This was most evident in the BST2-transfected PPRV infected cells where there was clear perturbation of H trafficking in areas of infected cell syncytia expressing larger levels of the overexpressed BST2 protein. Given the importance of BST2 in determining virus host susceptibility and disease pathogenesis [42] the cross specificity of mammalian BST2 proteins against morbillivirus H proteins is of interest and an area for continued investigation.

Our studies focused on the overexpression of BST2 in vitro highlighting specific dysregulation of the morbillivirus H protein. Further work is required to examine the effect of endogenous BST2 on morbillivirus glycoprotein activity, at baseline or IFN-induced levels, and this is the focus of ongoing work in our laboratory. In addition, it remains to be determined what effect BST2-specific inhibition of morbillivirus H proteins has on viral infection in vivo. Intriguingly, although Holmgren et al. demonstrated upregulation of BST2 (via a type I IFN response) in primary murine neurons and the brains of intracranially infected mice, its removal, in related KO mice studies, had no effect on pathogenesis [9]. This may be due to a MeV-vaccine-specific phenotype or, alternatively, a reflection of the built-in redundancy of the innate immune response following type I induction. Given the complex bitropic life cycle of morbilliviruses in SLAMF1-positive immune cells and nectin-4 positive epithelia, the role of restriction factors, including BST2, in the innate response to infection is an area of increasing interest. Our research also highlights the advantage of using quantitative assays modeling aspects of the viral life cycle, e.g., the MeV vGPs cell–cell fusion assay to characterize such restriction factors.

## Supplementary Materials

The following are available online at https://www.mdpi.com/1999-4915/11/8/692/s1, Figure S1: MeV entry is not inhibited by BST2, Figure S2: Incucyte analysis of MeV-GFP syncytia expansion, Figure S3: rLuc-GFP activity is not affected by BST2 over-expression.

## References

[1] World Health Orgnization Measles (Fact Sheet No 286). www.who.int/mediacentre/factsheets/fs286/en/.

[2] El Najjar F., Schmitt A.P., Dutch R.E. (2014). Paramyxovirus glycoprotein incorporation, assembly and budding: A three way dance for infectious particle production. Viruses.

[3] Muhlebach M.D., Mateo M., Sinn P.L., Prufer S., Uhlig K.M., Leonard V.H., Navaratnarajah C.K., Frenzke M., Wong X.X., Sawatsky B. (2011). Adherens junction protein nectin-4 is the epithelial receptor for measles virus. Nature.

[4] Ludlow M., McQuaid S., Milner D., de Swart R.L., Duprex W.P. (2015). Pathological consequences of systemic measles virus infection. J. Pathol..

[5] White R.G., Boyd J.F. (1973). The effect of measles on the thymus and other lymphoid tissues. Clin. Exp. Immunol..

[6] Podbilewicz B. (2014). Virus and cell fusion mechanisms. Annu. Rev. Cell Dev. Biol..

[7] Gotoh B., Komatsu T., Takeuchi K., Yokoo J. (2001). Paramyxovirus accessory proteins as interferon antagonists. Microbiol. Immunol..

[8] Zilliox M.J., Moss W.J., Griffin D.E. (2007). Gene expression changes in peripheral blood mononuclear cells during measles virus infection. Clin. Vaccine Immunol.: Cvi..

[9] Holmgren A.M., Miller K.D., Cavanaugh S.E., Rall G.F. (2015). Bst2/Tetherin Is Induced in Neurons by Type I Interferon and Viral Infection but Is Dispensable for Protection against Neurotropic Viral Challenge. J. Virol..

[10] Zilliox M.J., Parmigiani G., Griffin D.E. (2006). Gene expression patterns in dendritic cells infected with measles virus compared with other pathogens. Proc. Natl. Acad. Sci. USA.

[11] Neil S.J. (2013). The antiviral activities of tetherin. Curr. Top. Microbiol. Immunol..

[12] Neil S.J., Zang T., Bieniasz P.D. (2008). Tetherin inhibits retrovirus release and is antagonized by HIV-1 Vpu. Nature.

[13] Giese S., Marsh M. (2014). Tetherin can restrict cell-free and cell-cell transmission of HIV from primary macrophages to T cells. PLoS Pathog..

[14] Jia X., Weber E., Tokarev A., Lewinski M., Rizk M., Suarez M., Guatelli J., Xiong Y. (2014). Structural basis of HIV-1 Vpu-mediated BST2 antagonism via hijacking of the clathrin adaptor protein complex 1. eLife.

[15] Gustin J.K., Bai Y., Moses A.V., Douglas J.L. (2015). Ebola Virus Glycoprotein Promotes Enhanced Viral Egress by Preventing Ebola VP40 From Associating With the Host Restriction Factor BST2/Tetherin. J. Infect. Dis..

[16] Pan X.B., Qu X.W., Jiang D., Zhao X.L., Han J.C., Wei L. (2013). BST2/Tetherin inhibits hepatitis C virus production in human hepatoma cells. Antivir. Res..

[17] Pan X.B., Han J.C., Cong X., Wei L. (2012). BST2/tetherin inhibits dengue virus release from human hepatoma cells. PLoS ONE.

[18] Zenner H.L., Mauricio R., Banting G., Crump C.M. (2013). Herpes simplex virus 1 counteracts tetherin restriction via its virion host shutoff activity. J. Virol..

[19] Galao R.P., Pickering S., Curnock R., Neil S.J. (2014). Retroviral retention activates a Syk-dependent HemITAM in human tetherin. Cell Host Microbe.

[20] Hotter D., Sauter D., Kirchhoff F. (2013). Emerging role of the host restriction factor tetherin in viral immune sensing. J. Mol. Biol..

[21] Birch J., Juleff N., Heaton M.P., Kalbfleisch T., Kijas J., Bailey D. (2013). Characterization of ovine Nectin-4, a novel peste des petits ruminants virus receptor. J. Virol..

[22] Hashimoto K., Ono N., Tatsuo H., Minagawa H., Takeda M., Takeuchi K., Yanagi Y. (2002). SLAM (CD150)-independent measles virus entry as revealed by recombinant virus expressing green fluorescent protein. J. Virol..

[23] Frecha C., Costa C., Negre D., Gauthier E., Russell S.J., Cosset F.L., Verhoeyen E. (2008). Stable transduction of quiescent T cells without induction of cycle progression by a novel lentiviral vector pseudotyped with measles virus glycoproteins. Blood.

[24] Moll M., Klenk H.D., Maisner A. (2002). Importance of the cytoplasmic tails of the measles virus glycoproteins for fusogenic activity and the generation of recombinant measles viruses. J. Virol..

[25] Ishikawa H., Meng F., Kondo N., Iwamoto A., Matsuda Z. (2012). Generation of a dual-functional split-reporter protein for monitoring membrane fusion using self-associating split GFP. Protein Eng. Des. Sel.: Peds.

[26] Cathomen T., Naim H.Y., Cattaneo R. (1998). Measles viruses with altered envelope protein cytoplasmic tails gain cell fusion competence. J. Virol..

[27] Galao R.P., Le Tortorec A., Pickering S., Kueck T., Neil S.J. (2012). Innate sensing of HIV-1 assembly by Tetherin induces NFkappaB-dependent proinflammatory responses. Cell Host Microbe.

[28] Murphy L., Varela M., Desloire S., Ftaich N., Murgia C., Golder M., Neil S., Spencer T.E., Wootton S.K., Lavillette D. (2015). The sheep tetherin paralog oBST2B blocks envelope glycoprotein incorporation into nascent retroviral virions. J. Virol..

[29] Gnirss K., Fiedler M., Kramer-Kuhl A., Bolduan S., Mittler E., Becker S., Schindler M., Pohlmann S. (2014). Analysis of determinants in filovirus glycoproteins required for tetherin antagonism. Viruses.

[30] Vande Burgt N.H., Kaletsky R.L., Bates P. (2015). Requirements within the Ebola Viral Glycoprotein for Tetherin Antagonism. Viruses.

[31] Hauser H., Lopez L.A., Yang S.J., Oldenburg J.E., Exline C.M., Guatelli J.C., Cannon P.M. (2010). HIV-1 Vpu and HIV-2 Env counteract BST-2/tetherin by sequestration in a perinuclear compartment. Retrovirology.

[32] Blondeau C., Pelchen-Matthews A., Mlcochova P., Marsh M., Milne R.S., Towers G.J. (2013). Tetherin restricts herpes simplex virus 1 and is antagonized by glycoprotein M. J. Virol..

[33] Liu Y., Luo S., He S., Zhang M., Wang P., Li C., Huang W., Hu B., Griffin G.E., Shattock R.J. (2015). Tetherin restricts HSV-2 release and is counteracted by multiple viral glycoproteins. Virology.

[34] Narkpuk J., Wanitchang A., Kramyu J., Frantz P.N., Jongkaewwattana A., Teeravechyan S. (2014). An unconventional BST-2 function: Down-regulation of transient protein expression. Biochem. Biophys. Res. Commun..

[35] Goncalves-Carneiro D., McKeating J.A., Bailey D. (2017). The measles virus receptor SLAMF1 can mediate particle endocytosis. J. Virol..

[36] Ohno S., Ono N., Takeda M., Takeuchi K., Yanagi Y. (2004). Dissection of measles virus V protein in relation to its ability to block alpha/beta interferon signal transduction. J. Gen. Virol..

[37] Takaki H., Watanabe Y., Shingai M., Oshiumi H., Matsumoto M., Seya T. (2011). Strain-to-strain difference of V protein of measles virus affects MDA5-mediated IFN-beta-inducing potential. Mol. Immunol..

[38] Ohta K., Goto H., Yumine N., Nishio M. (2016). Human parainfluenza virus type 2 V protein inhibits and antagonizes tetherin. J. Gen. Virol..

[39] Nishio M., Garcin D., Simonet V., Kolakofsky D. (2002). The carboxyl segment of the mumps virus V protein associates with Stat proteins in vitro via a tryptophan-rich motif. Virology.

[40] Gnirss K., Zmora P., Blazejewska P., Winkler M., Lins A., Nehlmeier I., Gartner S., Moldenhauer A.S., Hofmann-Winkler H., Wolff T. (2015). Tetherin Sensitivity of Influenza A Viruses Is Strain Specific: Role of Hemagglutinin and Neuraminidase. J. Virol..

[41] Arnaud F., Black S.G., Murphy L., Griffiths D.J., Neil S.J., Spencer T.E., Palmarini M. (2010). Interplay between ovine bone marrow stromal cell antigen 2/tetherin and endogenous retroviruses. J. Virol..

[42] Weinelt J., Neil S.J. (2014). Differential sensitivities of tetherin isoforms to counteraction by primate lentiviruses. J. Virol..

