# Supplementary material for: BST2/Tetherin Overexpression Modulates Morbillivirus Glycoprotein Production to Inhibit Cell–Cell Fusion
Source: Viruses. 2019 Jul 30;11(8):692. doi: 10.3390/v11080692 (PMC6723339; doi:10.3390/v11080692)
Supplement: Supplementary file 1 [file viruses-11-00692-s001.pdf]

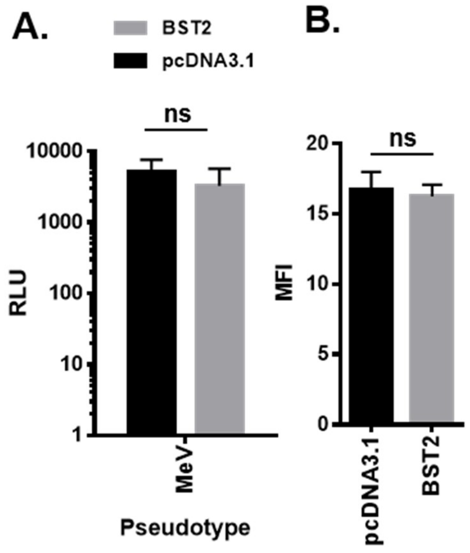

Supplemental Figure 1: MeV entry is not inhibited by BST2. (**A**) 293-hSLAM cells, transfected with the indicated pcDNA3.1-based expression constructs, were infected with MeV pseudotypes for 48 hours, before assessment of Firefly luciferase activity. (**B**) 293-hSLAM cells, transfected with the indicated pcDNA3.1-based expression constructs, were stained with an anti-SLAMF1 antibody (BD) and the mean fluorescence intensity (MFI) calculated by flow cytometry analysis.

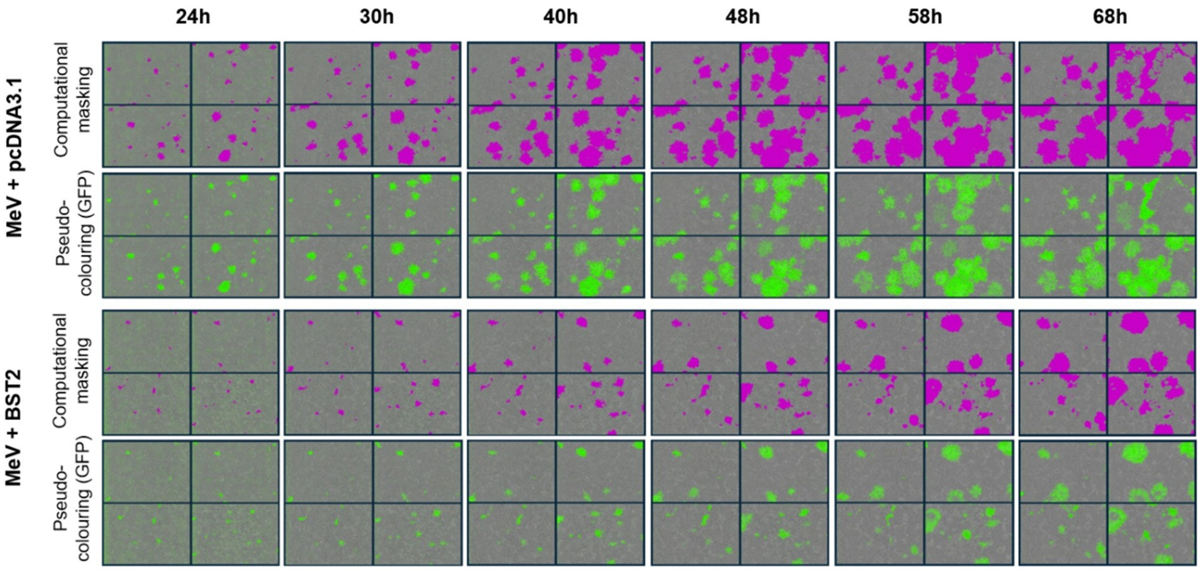

Supplemental Figure 2: Incucyte analysis of MeV-GFP syncytia expansion. Incucyte analysis proceeds by computational masking of coloured objects, informed by images taken under phase. In a process of machine learning, informed by selection of a limited number of images the software is trained to identify phase objects of specific size, and with certain accompanying fluorometric properties (e.g. intensity). Once achieved the analysis can then be run on an entire data set, providing a kinetic analysis of fluorophore expression over time,

in this case syncytia formation and expansion. A composite selection of images is chosen in this example with the top row showing the computationally masked objects and bottom row showing the more conventional pseudo-coloured GFP. In this instance the effect of BST2 expression on syncytia expansion can clearly be seen.

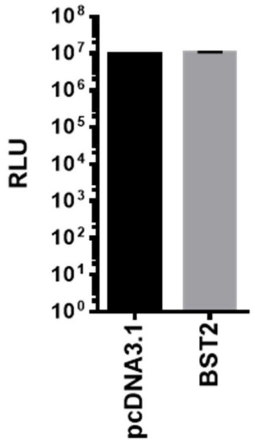

Supplemental Figure 3: rLuc-GFP activity is not affected by BST2 over-expression. HEK293T cells were co-transfected with both halves of the rLuc-GFP construct, as well as the indicated pcDNA3.1-based expression constructs, and the reporter activity measured 48 hours post-transfection.
